# Atlanta Academy of Medicine

**Published:** 1872-10

**Authors:** Jas. B. Baird


### ATLANTA ACADEMY OF MEDICINE.

JAS. B. BAIRD, M.D., REPORTER.

[Extract from Minutes, October 21st, 1872—Dr. J. P. LOGAN, President, in the Chair.]

By invitation, Dr. R. B. Anderson, of Roswell, read the following history of two cases which occurred in his practice, demonstrating, to his mind, the importance and practicability of turning the child in utero, and in many cases correcting a faulty position, by external manipulation:

### *TURNING A CHILD BY EXTERNAL MANIPULATION.*

Some time during the year 1870, I was called to see a colored woman in labor, about forty-three years of age, the mother of several children. Upon examination, I found the os uteri dilated, and the sack of waters protruding, but could not reach the child. I then examined the abdomen, and found the head in the right side of the mother. In the absence of pain, with one hand I pressed the head downward, at the same time pressing up with the other hand in the left side until I succeeded in bringing the head down to, and rather under, the symphysis pubis. But strange to say, every pain had a tendency to turn the child back in its first position. Unfortunately, a friend of mine received a serious injury during the labor, and I requested

Dr. —— to take my place with the woman until I could see him, which was agreed to, and I left her for three or four hours. On my return, the Doctor informed me that he had not touched her during my absence. I again examined, and found a breech presentation, and so delivered her—mother and child both doing well. Could I have remained, I am confident the head could have been kept in the proper position, as I have so done in several cases since.

My last case was on the 15th of the present month—Mrs. L., aged about forty years; the mother of eight or ten children—three only are living, and but one out of all that she had was carried to full term. I saw her, as stated, on the 15th; found her in labor, os uteri well dilated, and the bag of waters protruding from the os. The parts, except the os, I found to be hot and dry, with rigid perinæum and some hemorrhage, which she said she never failed to have at the commencement of all her labors. She also complained of severe headache. I gave her thirty grains of bromide of potassium, and waited half an hour, when I again examined. By pressing up the bag of waters, I could just feel the tips of the fingers—I supposed of the right hand, from the position I found the child to be in upon further examination. Placing my hand upon the abdomen, I found the child to be directly across, the head to the right side of the mother, and the knees pressing against the left side of the abdomen. On placing my hand upon the left side, she exclaimed: "Oh! Doctor, you will kill me; that place is as tender as my eye, and has been for several days." I handled her as carefully as possible, pressing the knees up with one hand, and at the same time pressing the head down toward the symphysis. I soon succeeded in bringing the head down under the symphysis pubis, and the next pain demonstrated the fact that all was right so far as related to presentation. The tenderness left the side, and she became quiet, having but little labor-pain for some time, but still complained of her head. I again gave her thirty grains of bromide of potassium, and in one hour her head was quite relieved. Labor progressed rather slowly, in consequence of the rigid perinæum, which at last yielded, and she was safely delivered of a seven months' child.

I could present several other cases of like character that have occurred in my practice, but think it unnecessary. In conclusion, I will just add, that I have never attempted to turn the child in this way after the waters had escaped.

Dr. W. F. Westmoreland had no practical experience with the method proposed by Dr. Anderson, but had no doubt that it could often be rendered serviceable.

Dr. S. H. Stout thought that common sense would dictate this mode of procedure, and he had no doubt but that it was frequently done. He always made use of the child as a lever, by placing one hand on the abdomen of the mother, in the process of turning.

Dr. Anderson reported a case in which the feet presented. He held them in his hand. Gave the woman forty grains of bromide of potassium; and after two hours' sleep, the head presented, and the child was delivered.

Dr. Stout suggested that at first the feet might have presented by the right side of the head.

Dr. James. B. Baird reported the following:

*A CASE OF DISEASE IN THE ARTERIES OF THE BRAIN, CAUSING SOFTENING OF THE BRAIN AND DEATH.*

Mary McR., colored, æt. forty-four years, a widow, washerwoman by occupation; has been troubled more or less for ten years past with rheumatism, having had, during that time, several attacks of acute articular rheumatism; she frequently complained of a numbness and a sensation of weakness in her extremities, particularly of the left side. About the 20th of September last, she received a fall; in attempting to descend a flight of stairs leading to the pavement, she tripped, and rolling down the steps, fell on the stones in the street. She was stunned for a few moments, and received several bruises upon her face, neck, and various portions of her body. There was also a slight incised wound of the upper lip, produced by her teeth, which healed kindly. The injuries sustained by the fall were very trifling, however, and she recovered from them in a few days.

I was called to see her, October 2d, and was informed that on the preceding Saturday evening, while sitting in a chair, she exclaimed suddenly that she had "lost the use of herself," and fell to the floor. She was picked up and placed upon her bed. She did not lose consciousness, though her nurse thought that she was occasionally slightly incoherent throughout the attack. Upon examination, I found complete paralysis of the left side to exist—power of motion being lost in all of the muscles of the left side of the face, except, perhaps, in the orbicularis oculi, and in the tongue, and the upper and lower extremity. Sensation, though greatly diminished, was not entirely lost. When the surface of the skin was irritated, she would refer it to another portion of the body than that to which the irritation was applied. The temperature of the affected side was considerably diminished. She articulated with great difficulty, and very indistinctly. The saliva flowed constantly from her mouth, as deglutition was very imperfectly performed. Passed her urine throughout without difficulty. Pulse good—eighty per minute; respiration normal; temperature not taken.

*Diagnosis.*—Hemiplegia, from cerebral embolism, in the right hemisphere.

With the exception of a mercurial cathartic on the first day, to relieve long-standing constipation, and an occasional opiate, to allay restlessness, she received no medical treatment. She was nourished by the free injection, per rectum, of fresh milk, milk punch, and animal broths. Ice and iced water were freely allowed, though her nurse assured me that she did not swallow as much as a table-spoonful of anything in the twenty-four hours. Hunger, of which she complained greatly at first, seemed to be appeased by the injection of nutritive material into the bowel. She died at eleven o'clock A.M., October 16—two weeks from the day I first saw her.

*Autopsy, Four Hours after Death.*—Heart, lungs, and all of the thoracic viscera sound; intra-cranial lesions. The meninges presented an appearance of intense congestion, all of the vessels on the surface of the brain being turgid. The membranes contained about two ounces of clear fluid. In the right hemisphere, very extensive softening existed—at least three-fourths of the medullary substance of the right side of the cerebrum presenting a homogeneous appearance, and a semi-solid, *mushy* consistency. The arteries at the base of the brain presented a diseased appearance. The outline was rendered irregular, and to the unaided eye, numerous thrombi were distinguishable through the coats of the vessels, encroaching upon the area of the canal, and almost, at some points, completely occluding it.

Dr. Raushenberg, who saw the case several times before death, examined carefully the diseased arteries, and subjected the thrombi to microscopical examination, and he has kindly consented to furnish the results of his investigation.

I am indebted to Drs. Raushenberg, Armstrong and Simpson for their presence and generous assistance during the post-mortem examination.

Now, I believe that this softening had been progressing for a considerable time; that it was occasioned by a diminished supply of arterial blood, caused by the obstructions projecting from the walls of the arteries; and that the sudden attack of paralysis was due to the breaking down of the already softened cerebral substance, rather than to any sudden and complete stoppage of the circulation within the brain.

The following description of the condition of the arteries at the base of the brain was given by Dr. Raushenberg, who presented for inspection the circle of Willis, and microscopical preparations of the thrombi:

A closer examination of the base of the brain revealed globular enlargements, with pale-yellow discolorations of the arteries at different localities. The most prominent one of these yellow nodules was plainly visible at the junction of the vertebral arteries, investing the right vertebral one-third of an inch; smaller ones existed—one in the basilar, where the anterior cerebellar joins it, then at the junction of the two posterior cerebral arteries, and a similar but more bluish-colored swelling, half an inch long, within the right middle cerebral artery, exterior to where the right posterior communicating artery joins it.

In dividing the walls of the right vertebral artery longitudinally, at the largest yellow-covered swelling mentioned above, the lumen of the artery was filled out by a conical thrombus, with a central canal of the size of a very fine thread. The walls of the thrombus, microscopically examined, proved to consist of layers of elastic, fenestrated inner arterial membrane—the soft bulb between them of more or less degenerated connective tissue, numerous fat globules, and cholesterin crystals. Very small, single-contoured round cells, in lively, vibrating, forward motion, invested the connective tissue in such large numbers, that there was not the smallest open space left; and it appeared as if these organisms were each one engaged in finding, with difficulty, a passage along the interstices of the tissue. Outside of the connective tissue, amongst the fat globules and crystals of cholesterin, the same cells were seen—less crowded, but still very numerous—floating about in shifting currents. A very few short, staff-like cells were also seen.

Thus, it is evident that these yellow-colored nodules in the arteries of this specimen represent the atheromatous pustules and the thrombus spoken of—the true arterial atheroma of pathological anatomists.

A section and examination of the pustule at the junction of the posterior cerebral arteries showed the same state of things. A section of the arterial walls of the middle cerebral artery, at the enlarged and discolored spot spoken of above, showed the exterior and interior membranes considerably thickened—leaving but a very fine canal for the passage of the blood—and in a semi-cartilaginous condition, but no soft bulb or thrombus, as in the other arteries. This semi-cartilaginous thickening of the arteries is like the arethoma, one of the consequences of chronic inflammation of the arterial membranes, which, as is generally the case in the aorta, seems to occur also in the brain, principally at the divisions of the arteries.

Dr. Stout referred to a new operation of so-called "Normal Ovariotomy," by a distinguished surgeon, for whom he had a high regard. He reviewed the operation in its moral, legal and professional aspect, condemning it in strong terms, and affirmed that he could not imagine any combination of circumstances that would render this operation justifiable. It became necessary occasionally to remove diseased ovaries, to save the life of the sufferer, but healthy ovaries should never be removed until every means of restoring the patient had been exhausted; and these are inexhaustible. Send the patient to the mountains—on a sea-voyage; give her tonics, anti-spasmodics, and the so-called anti-phrodisiacs. He referred, in this connection, to the value of assafœtida and camphor. Bring moral influences to bear upon the woman; remove every cause of irritation and excitement; in short, improve her hygiene, and he believes that every case would be cured without resort to a formidable and *unsexing* operation.

The Doctor also referred to a tendency on the part of surgeons to throw the responsibility of a severe operation upon the sufferer or his or her friends. He thought the practice of consulting non-professional persons on these subjects reprehensible in the last degree. He then proceeded, at considerable length, to advocate the advisability of consulting and assisting nature more, and a less ready resort to art—always remembering and keeping in view the wonderful power of the organism to adapt itself to abnormities, and overcome disabilities growing out of them.

Dr. W. F. Westmoreland was obliged to take issue with Dr. Stout. He differed with him *in toto*. He regarded the operation, under certain circumstances, as not only justifiable, but it might be the duty of the surgeon to advise and even urge it as a means of alleviating suffering and saving life. So far as consulting with non-professional persons was concerned, he regarded it the duty of the surgeon to explain to the patient and his friends the danger attendant upon an operation, before commencing it. The most trifling operation is attended with some risk. The surgeon does not thus rid himself of the responsibility; he *can not* do so.

Dr. J. T. Johnson did not see any difference between removing an ovary for disease in itself, and extirpating a healthy ovary which was the *cause* of disease, and which rendered death preferable to life.

Dr. W. A. Love cited the case of a woman, reported before to the Academy, who was entirely destitute of a uterus. The vagina is represented by a cul-de-sac. This woman has been troubled for years with the regular return of the menstrual molimen, suffering greatly at every catamenial period, until her health has been seriously impaired. Everything that could be devised has been done for her, without avail. It would be absurd to expect in this case that nature would relieve. It is impossible for nature to do her part in the absence of the uterus. He regards the operation as an advance in surgery.

Dr. Stout advocated bleeding from the arm, with the old-fashioned thumb-lancet, in such a case, during or immediately preceding the menstrual molimen, even though the patient be so feeble that she can not sit up, and apparently on the very verge of death; and in the interval between her attacks, attend to her hygiene.

Dr. Love had resorted to general blood-letting, among the innumerable expedients adopted for the relief of his patient, with no benefit.

---

Differential Diagnosis of Anæmic from Organic Murmurs of the Heart.—Dr. James H. Hutchinson (*Philadelphia Medical Times*, August 15, 1872), in a lecture on anæmia, states that he has found a peculiarity in cardiac murmurs arising from anæmia, which is but obscurely alluded to by some writers on auscultation. The murmur will be found to be much more intense when the patient is in the recumbent position than when he is either standing or sitting. Having never failed to detect this greater intensity in the recumbent position in every instance in which he has auscultated anæmic patients, Dr. Hutchinson believes it is a characteristic of some importance in the differential diagnosis of anæmic from organic murmurs.—*Boston Medical and Surgical Journal.*

